# Japanese Mothers’ Intention to HPV Vaccinate Their Daughters: How Has It Changed over Time Because of the Prolonged Suspension of the Governmental Recommendation?

**DOI:** 10.3390/vaccines8030502

**Published:** 2020-09-03

**Authors:** Asami Yagi, Yutaka Ueda, Tatsuo Masuda, Sayaka Ikeda, Takashi Miyatake, Satoshi Nakagawa, Kei Hirai, Tomio Nakayama, Etsuko Miyagi, Takayuki Enomoto, Tadashi Kimura

**Affiliations:** 1 Department of Obstetrics and Gynecology, Osaka University Graduate School of Medicine, Suita, Osaka 565-0871, Japan; a.yagi@gyne.med.osaka-u.ac.jp (A.Y.); tmasuda@sg.med.osaka-u.ac.jp (T.M.); s.nakagawa@gyne.med.osaka-u.ac.jp (S.N.); tadashi@gyne.med.osaka-u.ac.jp (T.K.); 2 Center for Cancer Control and Information Services, National Cancer Center, Chuo-ku, Tokyo 104-0045, Japan; sayakaikeda0201@gmail.com; 3 Department of Obstetrics and Gynecology, Osaka Police Hospital, Tennoji-ku, Osaka 543-0035, Japan; tmiyatake@aim.com; 4 Department of Clinical Psychology, Graduate School of Human Sciences, Osaka University, Suita, Osaka 565-0871, Japan; khirai@grappo.jp; 5 Center for Public Health Sciences, National Cancer Center, Chuo-ku, Tokyo 104-0045, Japan; tomnakay@ncc.go.jp; 6 Department of Obstetrics and Gynecology, Yokohama City University Graduate School of Medicine, Kanazawa-ku, Yokohama, Kanagawa 236-0004, Japan; emiyagi@yokohama-cu.ac.jp; 7 Department of Obstetrics and Gynecology, Niigata University Graduate School of Medical and Dental Sciences, Chuo-ku, Niigata 951-8510, Japan; enomoto@med.niigata-u.ac.jp

**Keywords:** HPV vaccine, Japan, suspension of governmental recommendation, mother

## Abstract

The trend for cervical cancer in younger women has been increasing recently in Japan. However, as a result of the suspension of governmental recommendation, Japan’s HPV (human papillomavirus) vaccination rate for girls born since 2000 has dropped sharply. We conducted an internet survey in December of 2019, 76 months after the suspension of recommendation, to verify the intention of mothers to inoculate their daughter under current circumstances and compared with our previous surveys and leaflet intervention effect. The rates of mothers who replied that they would “inoculate” were significantly higher at 9 and 23 months, but by 32 months after the suspension the rate was significantly lower (*p* < 0.05, *p* < 0.05, *p* < 0.05, respectively). The rates of the mothers who replied they would not inoculate were significantly lower at 9 months and 23 months, but at 76 months was significantly higher (*p* < 0.05, *p* < 0.05, *p* < 0.05, respectively). We found that intervention with a leaflet that could be used under the current suspension of the governmental recommendation did not increase the mothers’ intention to inoculate their daughters. A leaflet that actively encourages vaccination may increase the intent of vaccination. It is strongly recommended that the MHLW promptly resume its recommendations for HPV vaccination.

## 1. Introduction

The incidence of cervical cancer is one of the few cancers that could potentially be almost entirely eliminated by current medical practices, that the WHO (World Health Organization) has made its Draft Global Strategy for achieving that lofty goal one of its global public health policies. “Elimination” in this case would mean that the age-adjusted incidence rate of cervical cancer would fall to less than four reported cases per 100,000 women. By the time of this publication, this WHO Policy Proposal should have been approved by the World Health Assembly (May 2020, check later). WHO’s Draft Global Strategy has three main pillars, “Prevent, Screen and Treat”. These emphasis points capture WHO’s comprehensive approach to cervical cancer that includes sustained cancer prevention efforts, early and effective screening for the cancer, affordable and achievable optimal treatment options for all pre-cancerous lesions, and, as a final effort, early diagnosis and aggressive, efficacious programs for the management of invasive cervical cancers [1]. To reach this ambitious goal of “elimination” by 2030, the following three global targets must be aligned and accelerated: First, a 90% coverage of Human Papillomavirus (HPV) vaccination of girls by their 15th year must be achieved; Second, 70% of women should be screened with high-performance tests for HPV infection and cervical cancer-related lesions by the ages of 35 to 45 years), and have a 90% treatment rate for any precancerous lesions; Third, management of 90% of invasive cancer cases.

Australia has already promoted several such national programs and has reported scenarios for cervical cancer elimination [2]. Other countries have also reported achieving a high coverage for HPV vaccination and reducing invasive cancer [3,4]. These countries will reach WHO’s goal of an incidence rate of less than 4 reported cases per 100,000 women and eliminate cervical cancer as a public health problem within the lifetime of today’s young girls.

In direct contrast, the status of Japan’s cervical cancer countermeasures has been disastrous and seemingly going in reverse. Since around 2000 the trend for cervical cancer in younger women has been increasing in Japan [5]. Especially, the number of adenocarcinomas that cannot be easily detected by cervical screening cytology has also been steadily increasing in our younger women [6]. Therefore, the number of women who suffer from cervical cancer, including simple intraepithelial lesions, before the birth of their first child, is increasing rapidly.

The subsidies local governments provided for the national HPV vaccination programs commenced in 2010; this became a routine public vaccination program in April of 2013. However, due to repeated news reports regarding so-called adverse post-vaccination events, the Ministry of Health, Labor and Welfare (MHLW) announced the “Suspension of its active inoculation recommendation for the cervical cancer prevention vaccine” in June of 2013 [7]. This suspension, now seven years since its announcement, remains in effect as of May 2020. As a result of the combined effects of repeated media reports of the alleged adverse events and MHLW’s consequential suspension of its active recommendation, Japan’s HPV vaccination rate for girls born since 2000 has dropped sharply [8]. Japan now lags conspicuously behind WHO’s strategy pillar of achieving a high national HPV vaccination rate. Although 17 academic associations including the Japan Society of Obstetrics and Gynecology and the Japan Pediatric Society have called for a restart of the recommendation of HPV immunization and published statements about it [9], the MHLW has continued with the suspension of the recommendation of routine immunization. The government seems to have put more weight on standing by the girls with diverse symptoms than being concerned about the future increase of cervical cancer [9].

The second strategy of WHO was for achieving a cervical cancer screening rate of 70%, The cervical cancer screening rate among Japanese women is currently roughly 40%. This 40% is low compared to the 70% to 80% rate in most/all other OECD countries, so the achievement of this target will be very difficult for Japan to reach [10,11].

We have previously conducted three different surveys on “the intention of mothers to inoculate their daughters with the HPV vaccine”. Our newest report discusses the results of our fourth such internet survey, conducted in December 2019. In particular, we reveal the changes over time in the mothers’ “awareness of the HPV vaccine”, “their awareness of news reports regarding the so-called “adverse vaccine events” and “the intention of mothers to inoculate their daughters with the HPV vaccine” by comparing the results of our four sequential internet surveys. In addition, we examined the effectiveness of supporting the mothers’ decisions by interventions using three types of leaflets. This survey comparison spans the period of 76 months since the announcement of the government’s suspension of its HPV-vaccination recommendation.

## 2. Materials and Methods

### 2.1. Methods for the Fourth Internet Survey

This latest internet survey was conducted in December of 2019, 76 months after the suspension of recommendation. The respondents were 1545 mothers who had HPV-unvaccinated daughters who were aged 12–16 (Table 1). Similar to our previous three surveys, representative groups were extracted from the available a very large pool of volunteer survey participants based on registered information that monitors pool member’s sex, age, household income, work status, and educational level and their answers from a screening survey. Using the screening survey, we checked for the existence of a daughter, living together with her mother, who was of the target age for the routine HPV vaccination program. If the respondents met these requirements, we first again asked them to confirm the age of their daughter and her HPV vaccination history. If the respondents had multiple eligible daughters, only answers for the eldest were collected. Following conducting the screening survey, we conducted the main survey of the mothers. We asked them several questions related to cervical cancer, the HPV vaccine, and the mothers’ intention to inoculate their daughters. Next, we randomly divided the mothers into three groups and displayed images of a group-specific leaflet on the internet survey screen (Figure S1). After they viewed the leaflet interventions, we re-asked them about their intention to inoculate their daughter, to get a before and after leaflet intervention result.

For the main survey, we analyzed for correlations between the following questions related to cervical cancer, the HPV vaccine, and the intention of the mothers to inoculate their daughters: (1) Knowledge of cervical cancer: Do you know that cervical cancer more often affects women in their 20′s and 30′s?, (2) Knowledge about the HPV vaccine: Do you know about the HPV vaccine? Do you know the HPV vaccine can prevent cervical cancer? Do you know that there were news reports regarding the so-called adverse events of the HPV vaccine, such as chronic pain and motor impairment, which were discussed in relation to HPV vaccination in the Council of the MHLW? [12] (3) Health behavior: Have you ever had cervical cancer screening? Have you ever received an influenza vaccine? (Table 2).

The contents of the three different leaflets that we presented on the screen are shown in Figure S2. The control group (Group 1) was shown MHLW’s current information leaflet. For Groups 2 and 3, we intervened with one or the other of two leaflets that we had created for this purpose. One leaflet was positively informative but was not regarded as an active recommendation of HPV vaccination, so theoretically it could be used even under the current government’s suspension of its recommendation (Group 2). The third leaflet we created actively recommends HPV vaccination, so it couldn’t be used under the current government’s suspension of its recommendation (Group 3). Both these latter leaflets mainly described the seriousness of cervical cancer, the beneficial effect of the HPV vaccine, and its overall safety, but the content of each differed somewhat, as described above. The actual images presented are shown in Figure S3, which is only displayed in the Japanese format.

The following two questions were compared to ascertain how many mothers who had answered “Unsure” or “Won’t inoculate” changed their intention to inoculate because of the leaflet intervention: (1) Would you inoculate your daughter with the HPV vaccine under the current circumstances? (2) Would you inoculate your daughter if the MHLW restarted its recommendation? In addition, we asked the following question to examine the factors that better promote inoculation under the recommendation suspension: While the MHLW has not yet restarted its recommendation, would you inoculate your daughter if any of the following events occur?—A recommendation by the family doctor, a recommendation by the school, a recommendation by local government, or an inoculation of a daughter’s friend.

### 2.2. Comparison with Our Previous Surveys.

We compared our current survey results with the internet surveys we had conducted at 9 months, 23 months, and 32 months after the initial suspension of recommendation [13,14,15]. Taking mothers who had HPV-unvaccinated daughters as the subjects, the past three internet surveys were conducted among 200 mothers the first time, 2060 mothers the second time, and 2000 mothers the third time, using the same general survey methods each time. We compared the answers regarding “awareness of HPV vaccine”, “awareness of the news reports regarding so-called adverse events” and “intention of mothers to inoculate their daughters with the HPV vaccine”. In addition, the mother’s preconditions imposed on the inoculation were compared with the second survey, which was conducted in the same way.

### 2.3. Statistics

Univariate analysis (chi-square test and residual analysis) was used for the statistical analysis. The level of statistical significance was set at *p* < 0.05. In comparison between multiple groups, Dunnett’s test was conducted, where the significant difference was set to *p* < 0.025 in comparison between the control group and the two test groups.

### 2.4. Ethical Statement

This study was approved by the Institutional Review Board and Ethics Committee of the Osaka University Medical Hospital (14361-9).

## 3. Results

### 3.1. Result of the Fourth Internet Survey

#### 3.1.1. Study Targets and Participants’ Characteristics

The characteristics of the internet survey respondents are shown in Table 1. Their educational background matched that of the National Census [16]. No differences were observed in the background of the three groups. Similarly, the background of the most recent respondents was not significantly different from the participants of the previous three surveys.

#### 3.1.2. Correlation between Question and the Intention of Mothers to Inoculate Daughter Under Current Circumstances

Regarding the collective mothers’ responses—before viewing our leaflet interventions, 11.1% (171/1545) had at first replied that their intentions regarding their daughters were to “inoculate under the current circumstances” (Table 2). Meanwhile, there were five times as many mothers (51.5%, 796/1545) who, before viewing their group’s leaflet, had initially responded that “they wouldn’t inoculate”.

The percentage of mothers who knew about cervical cancer, the HPV vaccine, and that the HPV vaccine could prevent cervical cancer was significantly higher in the group of mothers who initially replied that they would not inoculate their daughters than in the other groups (*p* < 0.05, respectively). The percentage who had no knowledge of cervical cancer or who had no knowledge of the HPV vaccine, or was significantly higher in the group who replied “unsure” than for the other two groups (*p* < 0.05, respectively). Furthermore, regardless of the pre-intervention intention of the mothers to inoculate, each rate of the mothers who were aware of cervical cancer, the HPV vaccine and the effects of the vaccine were high: 82.6% (1276/1545), 91.7% (1416/1545), and 88.9% (1374/1545), respectively. The percentage of mothers who knew about the negative news reports regarding the so-called adverse events in the groups of mothers who replied they would “inoculate“ or “unsure” were significantly lower, while the rate of that in the group of the mothers who replied they would “won’t inoculate” was significantly higher: 91.0% (120/171), 80.3% (446/578), and 93.5% (774/796) (*p* < 0.05, *p* < 0.05, respectively). No correlation was observed with the mothers’ health behavior, including the mother’s previous cervical cancer screening pattern and influenza vaccination status, and their intention to inoculate their daughters.

#### 3.1.3. Verification of Leaflet Intervention Effect

**Figure 1:**
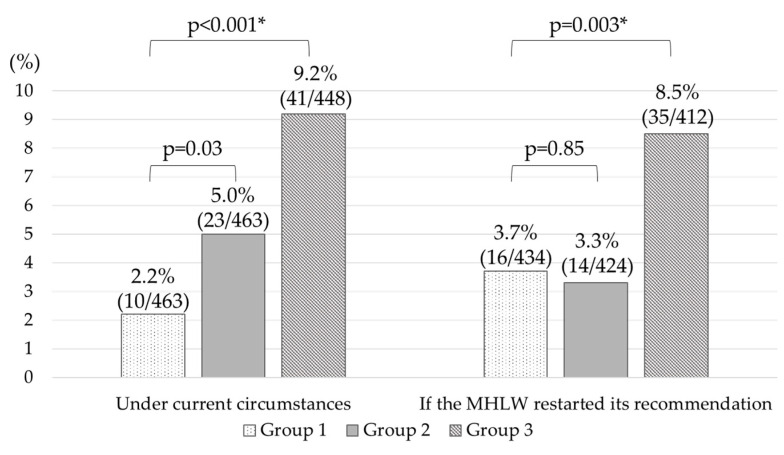
Leaflet intervention effect. In the mothers who had no intention to suggest vaccination to their daughter under the current circumstances, 9.2% (41/448) of Group 3 became newly willing to inoculate their daughters after intervention with our leaflets. This increase was significantly higher than the 2.2% (10/463) of Group 1 (*p* < 0.001). If the MHLW restarted their recommendation, 8.5% (35/412) of Group 3 turned willing to inoculate their daughters after intervention with the leaflets. Compared to 3.7% (16/434) of Group 1, the increase in the rate of Group 3 was significantly higher (*p* = 0.0037). * Significant difference was set to *p* < 0.025 due to a multiple comparison correction.

In the mothers who had no intention to suggest vaccination to their daughter under the current circumstances, 5.0% (23/463) of Group 2 and 9.2% (41/448) of Group 3 became newly willing to inoculate their daughters after intervention with our leaflets (Figure 1). Compared with 2.2% (10/463) of Group 1, the increased rate of Group 3 was significantly higher (*p* < 0.001, respectively). If the MHLW restarted their recommendation, 3.3% (14/424) of Group 2 and 8.5% (35/412) of Group 3 turned willing to inoculate their daughters after intervention with the leaflets. Compared to 3.7% (16/434) of Group 1, the increase in the rate of Group 3 was significantly higher (*p* = 0.0037).

#### 3.1.4. Factors That Would Promote Inoculation

The rates of the mothers who replied they would “inoculate” were significantly higher in the case of receiving a recommendation by the family doctor, even if the MHLW hadn’t restarted its recommendation in the hypothetical situation where the MHLW had restarted their recommendation. (*p* < 0.05, respectively) (Table 3).

### 3.2. Comparison of Current Results with Those of Previous Surveys

#### 3.2.1. Changes in Response to Each Survey Question Over Time

**Figure 2:**
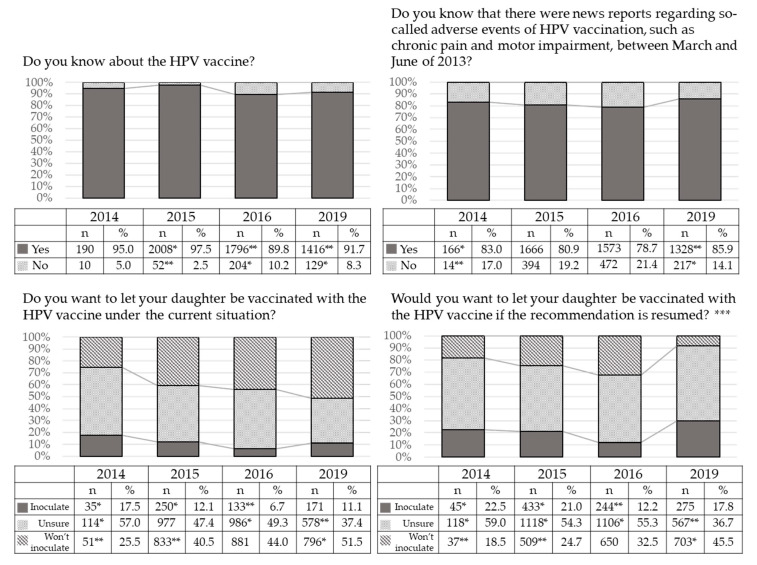
Changes in response to each survey question over time. The results of the present survey were compared to those of internet surveys we had conducted at 9 months, 23 months, and 32 months after the initial suspension of recommendation. The rates of mothers who replied that they would “inoculate” both under the current circumstances and under the hypothetical situation where the MHLW had restarted their recommendation were significantly higher at 9 and 23 months, but by 32 months after the suspension the rate was significantly lower (*p* < 0.05, *p* < 0.05, *p* < 0.05, respectively). The rates of the mothers who replied they wouldn’t inoculate were significantly lower at 9 months and 23 months, but at 76 months was significantly higher (*p* < 0.05, *p* < 0.05, *p* < 0.05, respectively). * Significantly higher, ** significantly lower. *p* < 0.05. *** The survey in Dec. 2019 didn’t ask this question of 200 people who didn’t know about the suspension of recommendation, so we used their answers from Q2 as the answer to this question.

The rate of the mothers who knew about the HPV vaccine was significantly higher at 23 months after the suspension, and those in the surveys responding after 32 and 76 months after the suspension were significantly lower (*p* < 0.05, *p* < 0.05, respectively), but at least 90% or more of the mothers knew about the HPV vaccine throughout the four surveys (Figure 2). The rate of the mothers who knew about the news reports regarding so-called adverse events was significantly higher at just 9 months after the suspension, and those in the surveys after 76 months was significantly faded (*p* < 0.05, *p* < 0.05, respectively), but about 80% of the mothers knew “about” the HPV vaccine throughout all the surveys.

The rates of mothers who replied that they would “inoculate” under the current circumstances were significantly higher at 9 and 23 months, but by 32 months after the suspension the rate was significantly lower (*p* < 0.05, *p* < 0.05, *p* < 0.05, respectively). The rates of the mothers who replied “unsure” were significantly higher at 9 and 32 months and that at 76 months was significantly lower (*p* < 0.05, *p* < 0.05, *p* < 0.05, respectively). The rates of the mothers who replied they would not inoculate were significantly lower at 9 months and 23 months, but at 76 months was significantly higher (*p* < 0.05, *p* < 0.05, *p* < 0.05, respectively).

The rates of the mothers who replied they would “inoculate” if the MHLW restarted their recommendation were significantly higher at 9 and 23 months, and that at 32 months after the suspension was significantly lower (p < 0.05, *p* < 0.05, *p* < 0.05, respectively). The rates of the mothers who replied “unsure” were significantly higher at 9, 23 and 32 months, but at 76 months was significantly lower (*p* < 0.05, *p* < 0.05, *p* < 0.05, *p* < 0.05, respectively). The rates of mothers who replied that they wouldn’t inoculate were significantly lower at 9 and 23 months, and that at 76 months was significantly higher (*p* < 0.05, *p* < 0.05, *p* < 0.05, respectively).

#### 3.2.2. Changes over Time in the Preconditions Mothers Would Require Before Allowing Their Daughters’ HPV Vaccination

**Figure 3:**
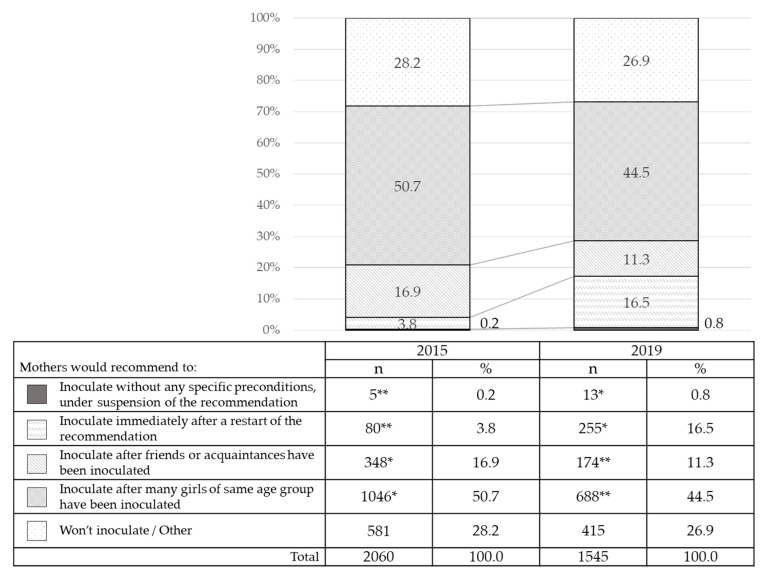
Changes over time in the preconditions mothers would require before allowing their daughters’ Human Papillomavirus (HPV) vaccination. The mother’s preconditions imposed on the inoculation in the latest survey were compared with the second survey conducted in the same way at 23 months after the suspension. The rates of the mothers who replied they would “inoculate without any specific preconditions” or would “inoculate immediately after a restart of the recommendation” were significantly lower at 23 months, and those at 76 months after the suspension were significantly higher (*p* < 0.05, *p* < 0.05, respectively). * Significantly higher, ** significantly lower. *p* < 0.05.

The rate of the mothers who replied they would “inoculate without any specific preconditions” or would “inoculate immediately after a restart of the recommendation” was significantly lower at 23 months, and that at 76 months after the suspension was significantly higher (*p* < 0.05, *p* < 0.05, respectively) (Figure 3). The rate of mothers who replied that they would “inoculate without any specific conditions” was less than 1% in all surveys, but the rate of the mothers who replied they would “inoculate immediately after a restart of the recommendation” increased by about four-fold. The rate of the mothers who replied they would “inoculate after friends or acquaintances have been inoculated” or “inoculate after many girls of same age group have been inoculated” was significantly higher at 23 months, but at 76 months was significantly lower (*p* < 0.05, *p* < 0.05, respectively). The rate of the mothers who replied that they would not inoculate even if many girls of the same age group have been inoculated, and the MHLW restarted their recommendation, was 28.2% at 23 months, and practically unchanged, 26.9%, at 76 months.

## 4. Discussion

In 2018, approximately 570,000 women developed cervical cancer worldwide, and 311,000 women died from this now easily avoidable disease [17]. It is estimated that in 2019, 10,500 people would suffer from cervical cancer in Japan, and 2900 would die of it [18]. Cervical cancer is a disease that is having a serious social impact in Japan, with Japan’s declining birthrate and its aging population. There is thus a critical impetus for a significant recovery of the nation’s HPV vaccination coverage, and that is an urgent goal that MUST be achieved as soon as possible.

Japan is not the only country whose vaccination rate had once dropped due to a deep public distrust of the HPV vaccine. In Ireland, the activity of anti-vaccine lobbying groups established in 2015 deeply reduced that nation’s HPV inoculation rate too, but in 2016 and 2017, first a steering group of concerned organizations, and then a pro-HPV Vaccination Alliance was established, forming powerful cross-sectoral alliances that rapidly led to improvements in HPV vaccine uptake [19]. Denmark has also recovered from its own imperiled vaccination coverage, in their case through a national information campaign about the HPV vaccine’s safety and effectiveness [20].

UNICEF has reported that the once novel HPV vaccine has now been introduced into 90 different countries [21]. Estimates of HPV vaccination coverage in 2018 in 58 countries, calculated using WHO methodology, were released in July of 2019. It was reported that 10 countries, including Malaysia, have already achieved an inoculation rate of 90% or higher, but, on the other hand, the inoculation rate is still less than 10% in five countries and that Japan has the lowest rate of vaccination. This data shows how other nations have grappled with the negative media coverage regarding HPV vaccination and how that negative impact can be countered with the steadying influences of action by national authorities, but it also raises questions as to what kinds of measures should be taken to best change the current impasse in Japan?

In our internet survey, we found that about 80 to 90% of the mothers surveyed were already aware of cervical cancer and the HPV vaccine and its effects, regardless of their own intentions for vaccination of their daughter (Table 2). The rate of mothers who knew about the news reports regarding the so-called adverse events in 2013, at around 80%, was also high. On the other hand, that rate decreased over time (Figure 2). Not that long after the MHLW suspension of its recommendation, the association between HPV vaccination and the alleged symptoms in young Japanese women mentioned by the media was scientifically refuted [22], but 51.5% of the mothers replied that they still wouldn’t inoculate their daughter under the current circumstances. This rate was significantly higher than in previous surveys, but the reasons for this increase are unknown. We are unaware of any new negative stories regarding the HPV vaccine circulating. The percentage of respondents who replied that they wouldn’t inoculate even if the MHLW restarted its recommendation was significantly higher in this most recent survey than in previous surveys. The most important new finding is that the rates of the mothers who were confused about inoculation were higher in past surveys; that level of confusion has decreased in this current study.

It is clear that Japanese mothers do not have low health awareness; 85.2% of the mothers had received both cervical cancer screening and an influenza vaccine (Table 2). However, most Japanese mothers still have heightened anxiety regarding the HPV vaccine. It is clear from our surveys that most Japanese mothers are choosing to avoid HPV vaccination—even though they also want to best protect their daughter’s health. It is thus important to provide them accurate information to dispel their anxiety about the HPV vaccine, but due to the suspension of recommendation, the wording in the information that is being provided at the national level is regrettably limited. In other words, local governments could even now be providing this beneficial information regarding the HPV vaccine, but must not yet be seen as openly recommending it.

We believe that the one leaflet we created is a positive recommendation for the vaccine, and that, shown by our survey, it could significantly increase the percentage of mothers intending to recommend that their daughter receive the HPV vaccination, both under the current circumstances and in a theoretical scenario where the governmental recommendation would be restarted (Figure 1). However, no increase in intention was observed in the group that was exposed to a leaflet we created that could be used under the current suspension of the MHLW recommendation. We have speculated that limitations placed on expressions of information permitted in the leaflets are one of the key factors that have suppressed any improvements in Japanese mother’s willingness to inoculate her daughter. The results suggest that the resumption of its recommendations is essential for the repromotion of the HPV vaccine. At the Council in January of 2020, regarding the matters of purpose and future direction of the information provided regarding the HPV vaccine, the MHLW approved a plan to shift in the direction of “Information provision should be implemented, regardless of local governments, as part of the public dissemination based on the Enforcement Ordinance of the Preventive Vaccination Law” [23]. With this new decision, individual delivery of information on HPV vaccination will be carried out by local governments, and improvement of information provision is expected.

What will happen to the HPV vaccination rate after such an information provision is improved? In the changes over time regarding the question of preconditions which mothers might require prior to their daughters’ HPV vaccination, the rates of the mothers who replied they would “inoculate without any specific conditions” or “inoculate immediately after a restart of the recommendation” were increased significantly (Figure 3). The rates of the mothers who replied they would “inoculate after friends or acquaintances have been inoculated” or “inoculate after many girls of same age group have been inoculated” were significantly lower, indicating a hardening of positions, both for and against. On the other hand, the rate of mothers who replied they “wouldn’t inoculate” did not change significantly. There is a possibility that the mothers’ tendency to make decisions, taking into consideration any surrounding circumstances, has all but disappeared. The rate of mothers who replied that they would “inoculate” if there was a recommendation from their family doctor was significantly higher. We believe that the family doctor has a major influence on vaccination decisions, so it is interesting that the attitudes of Japanese obstetricians and gynecologists regarding the HPV vaccine have recently been found to be improving. This changed position can be expected to provide a strong tailwind for improving our nation’s inoculation rate [24].

Simms et al. have made predictions regarding the lifetime cervical cancer morbidity and mortality rates in the population of young Japanese girls born between 1994 and 2007; their predictions were based on the current extremely low rate of HPV vaccination [25]. They predicted an increase of about 25,000 morbidities and about 5000 deaths, compared to a theoretical scenario of a continuous 70% vaccination coverage. We have also predicted an increase, of approximately 4000 in morbidities and 1000 in deaths per each school year in which girls were born after 2000 when the vaccination rate first dropped sharply (submitted).

We have already complained that the following efforts are needed as soon as possible: (1): Provide opportunities for immunization for younger women who are already older than the normally targeted ages of 12–16 years, this is because they were not immunized during the vaccine recommendation hiatus, and thus are at greater risk, (2): Start inoculating young boys, (3): Introduce the newer, more comprehensive nine-valent HPV vaccine, (4): Enhance local, national and public health recommendations for cervical cancer screening as a way to reduce the health damage incurred by the suspension of recommendation, (5): Create a special national action plan to re-promote HPV immunization, and (6): Provide more accurate and pro-vaccine information for the media to report [26]. We have begun to recognize the harsh reality that a girl born in Japan in the very near future may eventually die of cervical cancer as a direct result of MHLW’s suspension of its recommendation for HPV vaccination, and its decision to continue that suspension over these seven long years [8,27,28]. Medical institutions, educational institutions, governments, researchers, and the media should be working together hand-in-hand to reconsider the way they are each providing information to mothers—so that this tragedy-in-slow motion will not be repeated.

## 5. Conclusions

The rate of mothers who are confused about their daughters’ inoculation has decreased and that of mothers who would not inoculate has significantly increased in this current study. It was clear that a leaflet that could be used under the current suspension of the MHLW recommendation could not increase mothers’ intention to inoculate their daughters. These results suggested strongly that the resumption of recommendations is essential for the future re-emergence of the vaccine. The MHLW promptly should resume its recommendations for HPV vaccination.

## Figures and Tables

**Table 1:** Characteristics of the internet survey respondents.

	Group 1		Group 2		Group 3		*p*-Value	Total	
	*n*	%	*n*	%	*n*	%		*n*	%
Age									
30–39	87	16.9	92	17.9	88	17.1	0.95	267	17.3
40–49	373	72.4	364	70.7	365	70.9		1102	71.3
50 or older	55	10.7	59	11.4	62	12.0		176	11.4
Household income (Yen)									
Less than 4 million	95	18.5	110	21.4	94	18.3	0.57	299	19.4
4–6 million	114	22.1	121	23.5	124	24.1		359	23.0
6–8 million	94	18.3	80	15.5	79	15.3		253	16.4
Over 8 million	84	16.3	75	14.6	77	15.0		236	15.3
Don’t know	45	8.7	5	9.9	65	12.6		161	10.5
Did not reply to question	83	16.1	78	15.1	76	14.7		237	15.4
Work status									
Full-time job	97	18.9	115	22.3	100	19.4	0.64	312	20.2
Housewife	185	35.9	193	37.5	196	38.0		574	37.1
Part-time job	220	42.7	197	38.3	210	40.8		627	40.6
Other	13	2.5	10	1.9	9	1.8		32	2.1
Education level									
Junior High School/High School	172	33.4	192	37.3	162	31.5	0.39	526	34.0
Vocational School/Junior College	216	41.9	201	39.0	223	43.3		640	41.0
University/Graduate school	127	24.7	122	23.7	130	25.2		379	25.0
Total	515	33.3	515	33.3	515	33.3		1545	100.0

The respondents were 1545 mothers who had HPV-unvaccinated daughters who were aged 12–16. If the respondents had multiple eligible daughters, only answers for the eldest were collected. We randomly divided the mothers into three groups to display images of a group-specific leaflet on the internet survey screen (Figure S1) in the following surveys.

**Table 2:** Correlation between questions and intention of mothers to inoculate daughter under current circumstances.

	Inoculate		Unsure		Won’t inoculate		Total	
	*n*	%	*n*	%	*n*	%	*n*	%
Do you know that cervical cancer more often affects women in their 20s and 30s?								
Yes	147	86.0	450 **	77.9	679 *	85.3	1,276	82.6
No	24	14.0	128 *	37.4	117 **	14.7	269	17.4
Do you know about the HPV vaccine?								
Yes	155	90.6	508 **	87.9	753 *	94.6	1416	91.7
No	16	9.4	70 *	12.1	43 **	5.4	129	8.3
Do you know the HPV vaccine can prevent cervical cancer?								
Yes	154	90.1	491 **	85.09	729 *	91.6	1,374	88.9
No	17	9.9	87 *	15.0	67 **	8.4	171	11.1
Do you know that there were news reports regarding so-called adverse events of HPV vaccine, such as chronic pain and motor impairment, between March and June in 2013?								
Yes	120 **	91.0	464 **	80.3	774 *	93.5	1,328	86.0
No	51 *	29.8	114 *	19.7	52 **	6.5	217	14.0
Have you ever had cervical cancer screening?								
Yes	151	88.3	481	83.2	685	86.1	1,317	85.2
No	20	11.7	97	16.8	111	13.9	228	14.8
Have you ever received influenza vaccine?								
Yes	151	88.3	481	83.2	685	86.1	1,317	85.2
No	20	11.7	97	16.8	111	13.9	228	14.8
Total	171	11.1	578	37.4	796	51.5	1545	100.0

* Significantly high, ** significantly low, *p* < 0.05. Before viewing our leaflet interventions, 11.1% (171/1545) had at first replied that their intentions regarding their daughters were to “inoculate under the current circumstances”. Meanwhile, there were five times as many mothers (51.5%, 796/1545) who, before viewing their group’s leaflet, had initially responded that “they wouldn’t inoculate”. *: significantly higher (*p* < 0.05) by chi-square test and residual analysis. **: significantly lower (*p* < 0.05) by chi-square test and residual analysis.

**Table 3:** Factors that would promote inoculation.

	(MHLW Restarts Recommendation)		Recommendation by Family Doctor		Recommendation by School		Recommendation by Local Government		Inoculation of a Daughter’s Friend	
	*n*	%	*n*	%	*n*	%	*n*	%	*n*	%
Inoculate	(275/1545)	(17.8)	582/1545	37.7 *	445/1545	28.8	410/1545	26.5	478/1545	30.9

* Significantly high. *p* < 0.05. The rate of the mothers who replied they would “inoculate” in the case of receiving a recommendation by the family doctor even if the MHLW had not restarted its recommendation was 37.7% (582/1545), significantly higher than the 17.8% (275/1545) in the hypothetical situation where the MHLW had restarted their recommendation (*p* < 0.05). * Significantly higher. *p* < 0.05.

## Supplementary Materials

The following are available online at https://www.mdpi.com/2076-393X/8/3/502/s1, Figure S1: Survey flow, Figure S2: Leaflet contents, Figure S3: Image of leaflets (Only displayed in Japanese).
