# Supplementary material for: Japanese Mothers’ Intention to HPV Vaccinate Their Daughters: How Has It Changed over Time Because of the Prolonged Suspension of the Governmental Recommendation?
Source: Vaccines (Basel). 2020 Sep 3;8(3):502. doi: 10.3390/vaccines8030502 (PMC7577244; doi:10.3390/vaccines8030502)
Supplement: Supplementary file 1 [file vaccines-08-00502-s001.zip › Supplementary figure 1.pptx]

## Slide 1
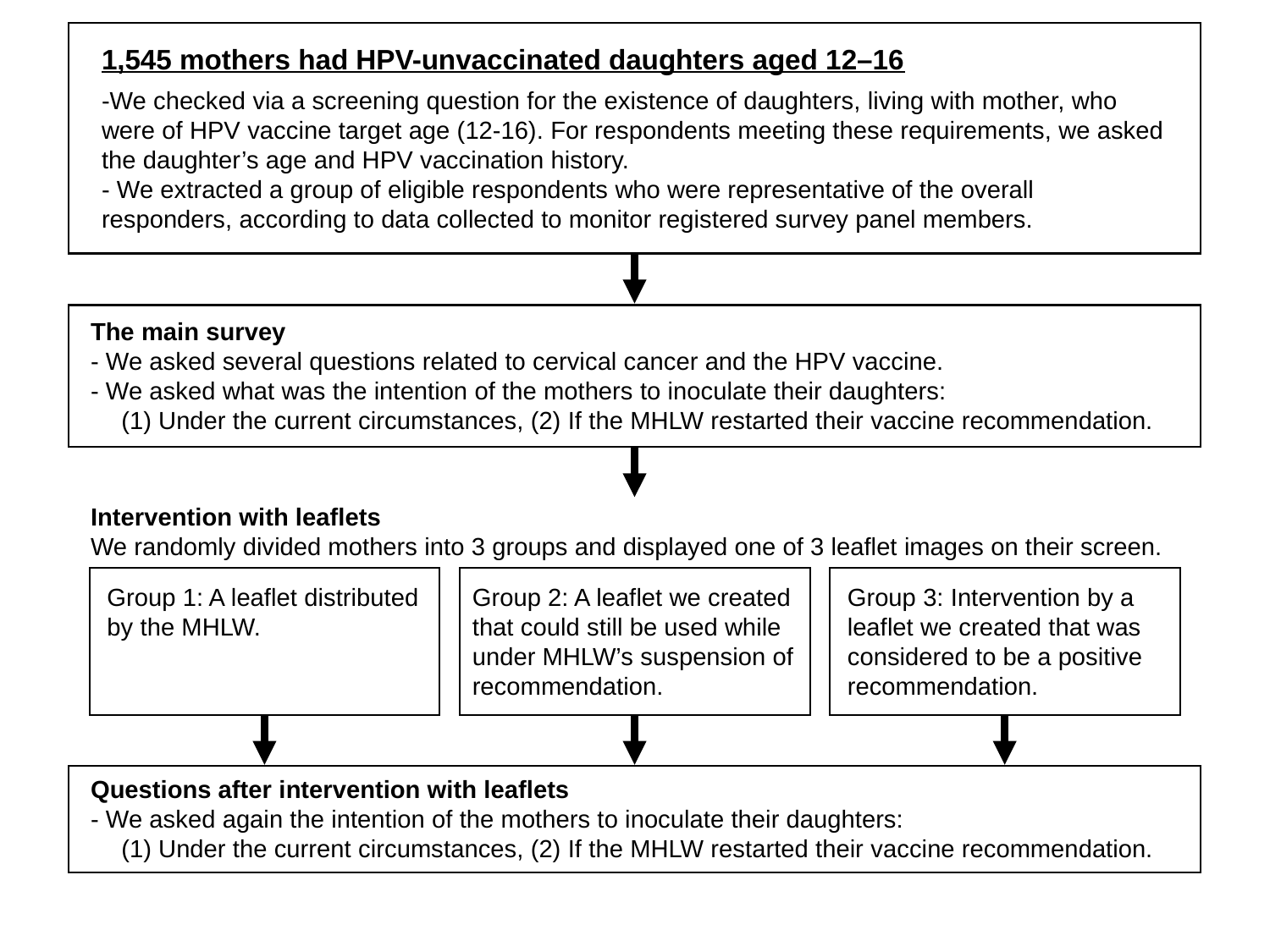

1,545 mothers had HPV-unvaccinated daughters aged 12–16
-We checked via a screening question for the existence of daughters, living with mother, who were of HPV vaccine target age (12-16). For respondents meeting these requirements, we asked the daughter’s age and HPV vaccination history.
- We extracted a group of eligible respondents who were representative of the overall responders, according to data collected to monitor registered survey panel members.
The main survey
- We asked several questions related to cervical cancer and the HPV vaccine.
- We asked what was the intention of the mothers to inoculate their daughters:
　(1) Under the current circumstances, (2) If the MHLW restarted their vaccine recommendation.
Intervention with leaflets
We randomly divided mothers into 3 groups and displayed one of 3 leaflet images on their screen.
Group 1: A leaflet distributed by the MHLW.
Group 2: A leaflet we created that could still be used while under MHLW’s suspension of recommendation.
Group 3: Intervention by a leaflet we created that was considered to be a positive recommendation.
Questions after intervention with leaflets
- We asked again the intention of the mothers to inoculate their daughters:
　(1) Under the current circumstances, (2) If the MHLW restarted their vaccine recommendation.
