# Supplementary material for: Japanese Mothers’ Intention to HPV Vaccinate Their Daughters: How Has It Changed over Time Because of the Prolonged Suspension of the Governmental Recommendation?
Source: Vaccines (Basel). 2020 Sep 3;8(3):502. doi: 10.3390/vaccines8030502 (PMC7577244; doi:10.3390/vaccines8030502)
Supplement: Supplementary file 1 [file vaccines-08-00502-s001.zip › Supplementary figure 2.pptx]

## Slide 1
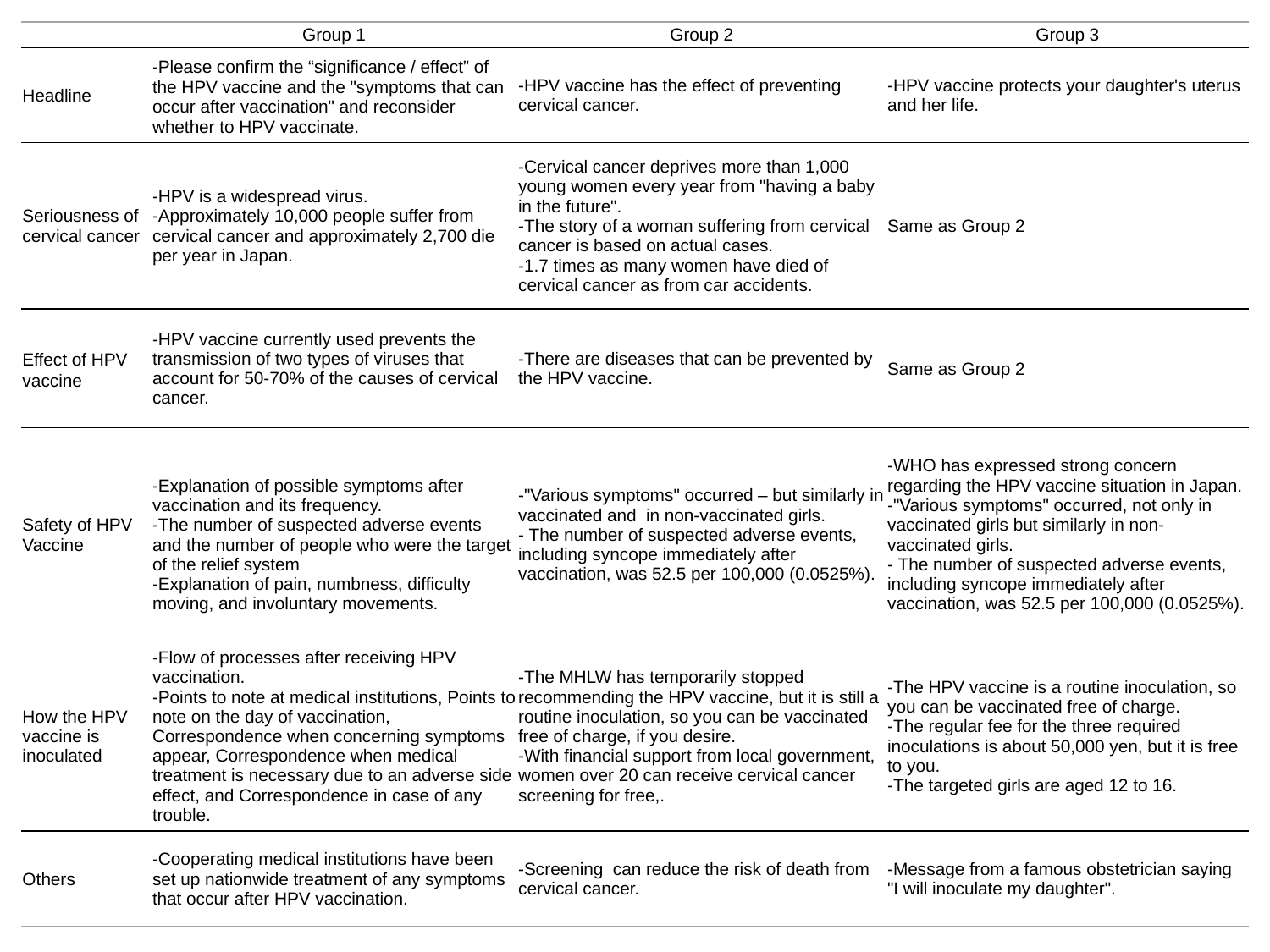

| | Group 1 | Group 2 | Group 3 |
| --- | --- | --- | --- |
| Headline | -Please confirm the “significance / effect” of　the HPV vaccine and the "symptoms that can occur after vaccination" and reconsider whether to HPV vaccinate. | -HPV vaccine has the effect of preventing cervical cancer. | -HPV vaccine protects your daughter's uterus and her life. |
| Seriousness of cervical cancer | -HPV is a widespread virus. -Approximately 10,000 people suffer from cervical cancer and approximately 2,700 die per year in Japan. | -Cervical cancer deprives more than 1,000 young women every year from "having a baby in the future". -The story of a woman suffering from cervical cancer is based on actual cases. -1.7 times as many women have died of cervical cancer as from car accidents. | Same as Group 2 |
| Effect of HPV　vaccine | -HPV vaccine currently used prevents the transmission of two types of viruses that account for 50-70% of the causes of cervical cancer. | -There are diseases that can be prevented by the HPV vaccine. | Same as Group 2 |
| Safety of HPV Vaccine | -Explanation of possible symptoms after vaccination and its frequency. -The number of suspected adverse events and the number of people who were the target of the relief system -Explanation of pain, numbness, difficulty moving, and involuntary movements. | -"Various symptoms" occurred – but similarly in vaccinated and in non-vaccinated girls. - The number of suspected adverse events, including syncope immediately after vaccination, was 52.5 per 100,000 (0.0525%). | -WHO has expressed strong concern regarding the HPV vaccine situation in Japan. -"Various symptoms" occurred, not only in vaccinated girls but similarly in non-vaccinated girls. - The number of suspected adverse events, including syncope immediately after vaccination, was 52.5 per 100,000 (0.0525%). |
| How the HPV vaccine is inoculated | -Flow of processes after receiving HPV vaccination. -Points to note at medical institutions, Points to note on the day of vaccination, Correspondence when concerning symptoms appear, Correspondence when medical treatment is necessary due to an adverse side effect, and Correspondence in case of any trouble. | -The MHLW has temporarily stopped recommending the HPV vaccine, but it is still a routine inoculation, so you can be vaccinated free of charge, if you desire. -With financial support from local government, women over 20 can receive cervical cancer screening for free,. | -The HPV vaccine is a routine inoculation, so you can be vaccinated free of charge. -The regular fee for the three required inoculations is about 50,000 yen, but it is free to you. -The targeted girls are aged 12 to 16. |
| Others | -Cooperating medical institutions have been set up nationwide treatment of any symptoms that occur after HPV vaccination. | -Screening can reduce the risk of death from cervical cancer. | -Message from a famous obstetrician saying "I will inoculate my daughter". |
