# Supplementary figures and images for: Japanese Mothers’ Intention to HPV Vaccinate Their Daughters: How Has It Changed over Time Because of the Prolonged Suspension of the Governmental Recommendation?
Source: Vaccines (Basel). 2020 Sep 3;8(3):502. doi: 10.3390/vaccines8030502 (PMC7577244; doi:10.3390/vaccines8030502)

## Slide 1
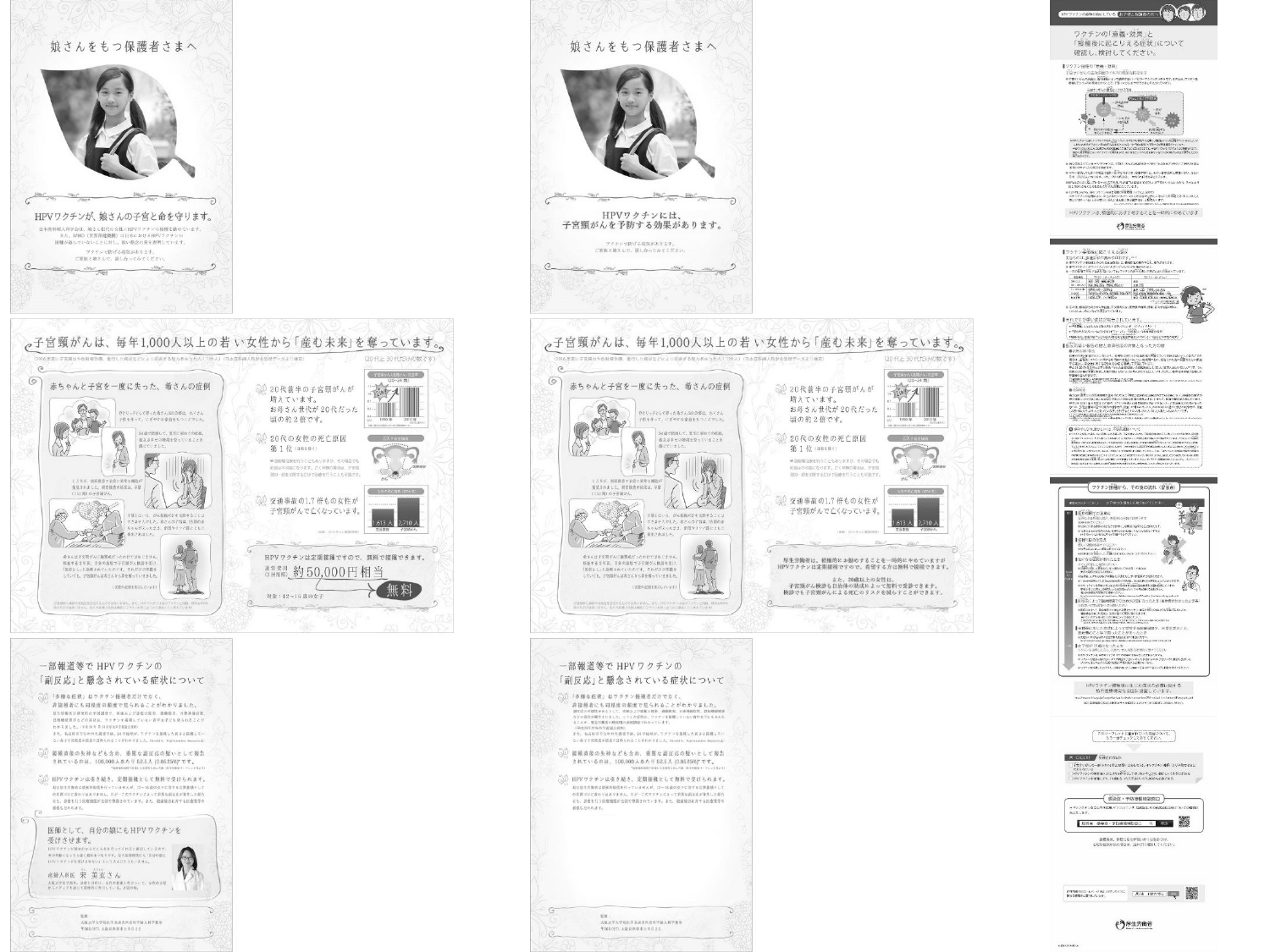

Supplement: Supplementary file 1 [file vaccines-08-00502-s001.zip › Supplementary figure 3.pptx]
